# *De novo* transcriptomic analysis of the alimentary tract of the tephritid gall fly, *Procecidochares utilis*

**DOI:** 10.1371/journal.pone.0201679

**Published:** 2018-08-23

**Authors:** Lifang Li, Mingxian Lan, Wufeng Lu, Zhaobo Li, Tao Xia, Jiaying Zhu, Min Ye, Xi Gao, Guoxing Wu

**Affiliations:** 1 State Key Laboratory for Conservation and Utilization of Bio-Resources in Yunnan, Yunnan Agricultural University, Kunming, China; 2 Key Laboratory of Forest Disaster Warning and Control of Yunnan Province, Southwest Forestry University, Kunming, China; Chinese Academy of Agricultural Sciences Institute of Plant Protection, CHINA

## Abstract

The tephritid gall fly, *Procecidochares utilis*, is an important obligate parasitic insect of the malignant weed *Eupatorium adenophorum* which biosynthesizes toxic secondary metabolites. Insect alimentary tracts secrete several enzymes that are used for detoxification, including cytochrome P450s, glutathione S-transferases, and carboxylesterases. To explore the adaptation of *P*. *utilis* to its toxic host plant, *E*. *adenophorum* at molecular level, we sequenced the transcriptome of the alimentary tract of *P*. *utilis* using Illumina sequencing. Sequencing and *de novo* assembly yielded 62,443 high-quality contigs with an average length of 604 bp that were further assembled into 45,985 unigenes with an average length of 674 bp and an N50 of 983 bp. Among the unigenes, 30,430 (66.17%) were annotated by alignment against the NCBI non-redundant protein (Nr) database, while 16,700 (36.32%), 16,267 (35.37%), and 11,530 (25.07%) were assigned functions using the Clusters of Orthologous Groups (COG), Kyoto Encyclopedia of Genes and Genomes (KEGG), and Gene Ontology (GO) databases, respectively. Using the comprehensive transcriptome data set, we manually identified several important gene families likely to be involved in the detoxification of toxic compounds including 21 unigenes within the glutathione S-transferase (GST) family, 22 unigenes within the cytochrome P450 (P450) family, and 16 unigenes within the carboxylesterase (CarE) family. Quantitative PCR was used to verify eight, six, and two genes of GSTs, P450s, and CarEs, respectively, in different *P*. *utilis* tissues and at different developmental stages. The detoxification enzyme genes were mainly expressed in the foregut and midgut. Moreover, the unigenes were higher expressed in the larvae, pupae, and 3-day adults, while they were expressed at lower levels in eggs. These transcriptomic data provide a valuable molecular resource for better understanding the function of the *P*. *utilis* alimentary canal. These identified genes could be pinpoints to address the molecular mechanisms of *P*. *utilis* interacting with toxic plant host.

## Introduction

*Eupatorium adenophorum* (Compositae: Eupatorium) is a vigorous perennial weed that is difficult to eradicate for its rapid growth and high reproduction rate [1, 2]. The weed originated in the United States, was introduced into Yunnan Province of China in 1940s, and then rapidly spread throughout other Southwestern regions, such as Sichuan, Chongqing, and Guizhou [3, 4]. Its spread has not only caused serious economic losses on agriculture, forestry, and animal husbandry sectors in China but also damaged habitat environments of native species [1, 5]. *E*. *adenophorum* is rarely infected by pathogenic bacteria and fungi or insects for containing abundant active plant secondary metabolites to defend against such attacks [6]. Such toxic substances could cause nausea, distended abdomens, indigestion, and other symptoms in livestock if accidental consumped [7, 8]. Moreover, mice, rabbits, and goats that are fed *E*. *adenophorum* can exhibit hepatomegaly, and even necrotic poisoning symptoms, and the plant’s pollen and seeds can cause asthma and lung tissue necrosisin animals [9–12]. Additionally, essential oil extracts from *E*. *adenophorum* can be used to control pests *Sitophilus oryzae*, *Sitophilus zeamais*, *Callosobruchus chinensis*, and *Bruchus rufimanus* [13] and are toxic to the sensory organs of *Myzus persicae* and *Lipaphis erysimi* [14].

The *Procecidochares utilis* Stone (Diptera: Tephritidae) is an important obligate parasite of *E*. *adenophorum* [15]. The adult fly deposit their eggs on the buds, shoot tips, and leaf veins of *E*. *adenophorum*. When the eggs hatch, the larvae bore into the tender stems of *E*. *adenophorum*, where they feed and stimulate gall formation and enlargement, resulting in the blockage of growth and decreased nutrient transport [16–18]. Thus, *P*. *utilis* plays an important role in the prevention and control of *E*. *adenophorum*. Although *E*. *adenophorum* can still grow with parasites, it’s the seed yield, plant height, branch number, photosynthetic capability, biomass accumulation, and distribution are all suppressed to affect its total growth [16, 19]. Investigations of *P*. *utilis* have extensively focused on its biology, ecology and potential control, however little is known about the genetic information of this fly.

Host plants and insects can be closely linked from physiological due to co-evolutionary relationships [20]. To withstand phytophagous insect-feeding, plants have morphological, biochemical, and regulatory defense mechanisms by long-term evolution [21]. Most plants rely on plant secondary metabolites to prevent from polyphagous insects. Plant secondary metabolites can deter feeding behaviors, inhibit insect growth and reproduction, as well as exhibit toxicity towards insects [22, 23]. Similarly, in the process of overcoming the deleterious effects and toxicity of plant secondary metabolites from their host plants, insects can develop adaptations towards plant secondary metabolites through several mechanisms, including feeding avoidance and/or the detoxification of plant secondary metabolites [21, 23, 24]. There are three important types of detoxification-related metabolic enzymes in insects: glutathione-S-transferases (GSTs), cytochrome P450 enzymes (P450s) and carboxylesterases (CarEs) [21, 25–27]. These enzymes play key roles not only in the detoxification-related metabolism of plant secondary metabolites but also in the adaptability of insects to host plants. Moreover, these enzymes are involved in the metabolism of insecticides [28–30]. *P450* genes belong to a large and diverse gene superfamily, whose members exhibit multifunctional activities and participate in the metabolism of endogenous substances and xenobiotics in most organisms [31–32]. Likewise, GSTs more widely detoxify insecticides and plant allelochemicals in insects and are a superfamily of enzymes widespread in most organisms [33–34]. GSTs can be classified into seven classes in insects, including the delta, epsilon, omega, sigma, theta, zeta, and microsomal groups, where the delta and epsilon classes are insect-specific [35–37]. Lastly, CarEs belong to a superfamily of metabolic enzymes that are generally distributed throughout the various parts of insects and play significant roles in the metabolic detoxification of xenobiotics, the degradation of hormones, in neurodevelopment, and in defense [38, 39].

The insect gut is the primary area for food digestion, ingestion, and utilization, and it is also the site for detoxification of plant secondary metabolites and insecticides [40]. Numerous P450s expressed in the midgut, hindgut, and Malpighian tubules have been identified in *Drosophila melanogaster* [31]. *Spodoptera litura* GSTs (delta, sigma, and theta groups) are highly expressed in midgut [41]. The expression levels of two CarE genes (*Pxae*22 and *Pxae*31) in the midgut of *Plutella xylostella* were significantly higher than in the head, cuticle, and fat bodies [42]. To assess the effect of plant allelochemicals on the expression of *P450* genes in *Manduca sexta*, Feyereisen (1999) found that increased expression of two P450s in the midgut, *CYP4M1* and *CYP4M3*, were induced by nicotine [43]. Taken together, the above studies indicated that a large number of detoxification enzymes in the midgut of insects, and the increased expression of these enzymes is related to the metabolism of exogenous toxic substances.

The entire life history of *P*. *utilis* completes in *E*. *adenophorum*, t which is the only e food source for parasite’s growth and development. *E*. *adenophorum* tissues are rich in toxic substances, including terpenoids, flavonoids, coumarins, sterols, alkaloids, and others [44, 45]. This suggests that during the process of adaptive evolution, the fly may have evolved mechanisms to detoxify the toxic secondary biomass of *E*. *adenophorum*. As indicated above, the digestive tract of insects is the primary organ that digests food and absorbs nutrients, but is also a major barrier to the toxic effects of plant secondary metabolites as well as pathogenic microorganisms.The digestive tract is thus important site for detoxification metabolism [46–47].

In this study, we generated a transcriptome for the digestive tract of *P*. *utilis* using the high-throughput Illumina HiSeq 2000 platform. Observed unigenes were annotated using several databases, and three major detoxification metabolic enzymes were identified from the GST, P450, and CarE families. The expression patterns of GST, P450, and CarE genes were further investigated in different tissues (epidermis, fat body, salivary gland, foregut, midgut, hindgut, and Malpighian tubules) and at different developmental stages (eggs, firstto third instar larvae, one to three day pupae, and male and female adults of one to five day age). The transcriptome data set provides a valuable molecular resource for future studies of *P*. *utilis* and investigation of the biological functions of GSTs, P450s, and CarEs in the *P*. *utilis* gut, particularly as they relate to detoxification metabolism.

## Materials and methods

### Ethics statement

The *P*. *utilis* used for this study is not involved in endangered or protected species. Galls of *E*. *adenophorum* were collected from the Chenggong District of Kunming, China and no special permission was required for this place, the galls of *E*. *adenophorum* used for this study is not involved in endangered or protected species.

### Insect samples

Galls of *E*. *adenophorum* were collected from the Chenggong District of Kunming, China. All galls were reared in cages, as described by Gao *et al*. (2014) [48]. A scalpel was then used to cut fresh galls, and larvae were collected for the experiments.

### Anatomy of the alimentary tract

The alimentary tracts of *P*. *utilis* third instar larvae were collected, including the foregut, midgut, hindgut, and Malpighian tubules. *E*. *adenophorum* larvae were first disinfected with 75% alcohol. The skins of larvae were then cut from head to tail using a dissecting needle. Fat body, cuticula, salivary glands, and other residues were removed and placed in phosphate buffered saline (PBS) solution on a concave glass slide under an anatomical lens. The dissected digestive tract was then washed three times with PBS on the concave slide and transferred to a centrifuge tube on ice containing TRIzol.

### Transcriptome library preparation and sequencing

Total RNA of the *P*. *utilis* alimentary tract was extracted using TRIzol (Invitrogen, Carlsbad, CA, USA) according to the manufacturer's instructions. RNA quantity and quality were assessed using an Agilent 2100 Bioanalyzer (Agilent RNA 6000 Nano Kit, Agilent Technologies, Palo Alto, CA, USA) and NanoDrop spectrophotometer (Thermo Scientific, Waltham, MA,USA), respectively.

RNA samples were sent to the Beijing Genomics Institute (BGI, Shenzhen, China) for cDNA library construction and Illumina sequencing. Briefly, poly (A) mRNA was purified from total RNA using magnetic beads and oligo (dT) and then fragmented into short fragments at 94°C for 5 min. First-strand cDNAs were synthesized from the RNA fragments using random hexamers. Then, second-strand cDNA synthesis was conducted using DNA polymerase I and RNase H. These cDNA fragments were washed and resolved with EB buffer for end repair and then ligated to sequencing adapters. Suitable fragments were then amplified by PCR to create the final cDNA library. The cDNA library was sequenced using paired-end sequencing on the Illumina HiSeq 2000 platform. Raw sequencing data were submitted to the NCBI Sequence Read Archive under the accession number: SRP136380.

### *De novo* assembly and bioinformatics analyses

Raw reads were filtered by removing adapter sequences, low-quality sequences with unknown nucleotides, and reads with more than 20% low-quality bases. The filtered transcriptomics data (i.e., the clean reads) were *de novo* assembled using the Trinity assembler [49]. BLASTX was used to functionally annotate the assembled unigenes using an E-value cut-off of 1e-5 and by comparing the unigenes to several databases including the NCBI-nr, NCBI-nt, SwissProt, Gene Ontology (GO), Clusters of Orthologous Groups of proteins (COG), and the Kyoto Encyclopedia of Genes and Genomes (KEGG) metabolic pathway databases. Genes were annotated based on the highest sequence similarity to gene models within the various databases. The best hits were used to determine the sequence direction of the unigenes. When different databases conflicted with each other, results were prioritized in the following order: nr, SwissProt, KEGG, and COG. Unigenes that did not match any of the above databases were further analyzed using the ESTScan software package to predict coding regions and the orientation of the sequences.

### Gene identification

To comprehensively identify P450, GST, and CarE genes, both BLAST2GO annotation and the Geneious (version 9.1.3) software package were used [50, 51]. First, the known amino acid sequences of P450s, GSTs, and CarEs from various insect species were retrieved from NCBI with the keywords “cytochrome P450 AND insecta”, “glutathione S-transferase,” and “carboxylesterase AND insecta”. All unigenes were then used to build a database in Geneious, and the collected P450, GST, and CarE sequences were compared (tBLASTn) against the Geneious database to identify unigenes. Finally, the unigenes identified by Geneious were compared against the NCBI database using BLASTX for further validation. The P450, GST, and CarE genes were then aligned with those from other insects. Finally, phylogenetic trees for the detoxification gene families were constructed using the neighbor-joining (NJ) method in the MEGA5 software package [52].

### QPCR

Total RNA was extracted from different tissues (cuticle, fat body, foregut, midgut, hindgut, Malpighian tubules, and salivary glands) and at different developmental stages (eggs, first to third instar larvae, one to three-day-old pupae and male and female adults one to five days after eclosion) using Trizol (Invitrogen) according to the manufacturer's instructions. Total RNA quality and quantity were assessed using 1% agarose gel electrophoresis and an UV-visible spectrophotometer, respectively. RNA samples were then incubated with DNase I (Takara, China) to remove genomic DNA contamination. Finally, 1 μg of RNA from each sample was used to synthesize cDNA templates using the RevertAid First Strand cDNA Synthesis Kit (Takara, China). Specific primers pairs were designed using the Primer Premier 6.0 software package (Table 1). The 18S ribosomal RNA gene was used as a reference. QPCR reactions using the SYBR Premix EX Taq (Takara) kit were performed in triplicate with three biological replicates in 96-well plates, following the manufacturer’s instructions. QPCR analysis was conducted on the StepOne Plus System (ABI Prism, Applied Biosystems). The following PCR conditions were used: 95°C for 30 s, followed by 40 cycles of 95°C for 5 s and 58°C for 40 s. Relative expression levels were determined using the2^−∆∆CT^ method [53]. Finally, data were analyzed using the SPSS software package (version 22).

**Table 1:** Primers used in QPCR.

Gene Name	Primer Sequences (Upstream primer/ Downstream primer) 5ʹ to 3ʹ
Unigene31129	GGTGAACACATGAAGCCGGAAT/TTGGGGTACAACGAATCATCCT
Unigene36303	ATTATGGGAATCACGGGCTAT/GGTTGACTACTGCTTGTTTGG
Unigene32289	TGCGAGATTGATCAACCAAAGT/ TCTCTGCCCTTTTCAATGTGT
Unigene21310	TGGTCAATCAATATGGACAGAGTC/TCGTCCCCACGTAATATACGAA
Unigene31382	CACATGCCATCAATGCCTATCT/CGCTCGAAAACTACTCCCGTAT
Unigene32072	GGGTAAACTGGTATTGTATGGGAT/ ATATTGACCGTCGTCTTCGAGT
Unigene27628	GGCTCATCGTAACGATTTGGA/CGACTGGGTTGTGGCATAACA
Unigene27629	TGCTAACACTCGAAAATGAAGTG/ TGAAAGTCTGCGTTCTGAAAC
Unigene929	CGTCATGGATATTGGCGTCG/TGCCAACAAATGGTGCTGTG
Unigene32932	GCACAACGCATGGGAAGATT/AAGCGCACATTTACTCCACC
Unigene1512	ACATGGAGATGGTTACGTCAGA/AATCCAACTACGGGAGTCCA
Unigene33767	GTATTTTCTTGCGATCCCGATG/CGGCCAGTACTCATAAGTAAACC
Unigene34353	TGGCTGGTGTTGATACGACAT/AGCGGATAGACACGAAATGCT
Unigene31805	TAGGGCCACGCATGTGCATT/TTACGTGGTACAAGGCGCAC
CL797.contig1	TGAGTGCATCACCATTGGGAC/GGCAATTCACTAACTCGACCG
18S rRNA	GCGAGAGGTGGAAATTCTTGG/ CGGGTAAGCGACTGAGAGAG

## Results

### Sequencing and *De novo* assembly

**Fig 1:**
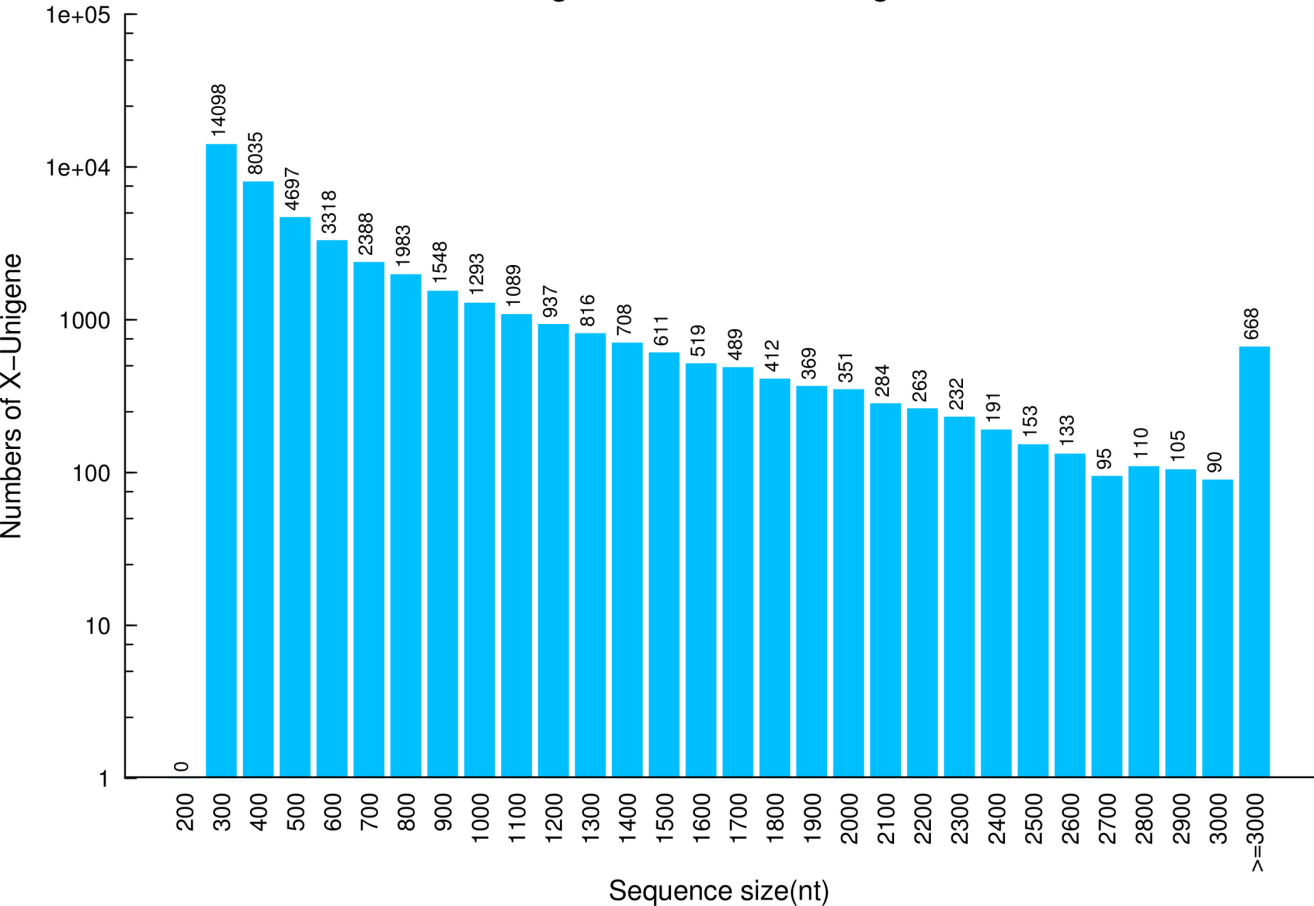
Length distribution of unigenes.

A transcriptomic library derived from the midgut tissues of *P*. *utilis* Stone larvae was constructed on the Illumina HiSeq 2000 platform in a single run that generated 7.75 Gb of raw data and comprised 94,204,900 raw reads. After quality filtering, a total of 86,124,048 clean reads were obtained (Table 2). The clean reads were *de novo* assembled into 62,443 contigs, with an average length of 604 bp. Finally, the reads were further assembled into 45,985 unigenes, with an average length of 674 bp. Of these unigenes, 19,155 (41.65%) were longer than 500 nt, 8,625 (18.76%) were longer than 1,000 nt, and 2,324 transcripts (5.05%) were longer than 2,000 nt (Fig 1).

**Table 2:** Summary of the *P*. *utilis* midgut transcriptome.

**Reads**	
Raw reads	94,204,900
Clean reads	86,124,048
Total Clean Bases (Gb)	7.75
Q20 percentage (%)	98.63
Q30 percentage (%)	93.77%
**Contigs**	
Total Number	62,443
Total Length (nt)	37,726,584
Mean Length (nt)	604
N50	894
**Unigenes (≥200 nt)**	
Total Number	45,985
Total Length (nt)	31,003,657
Mean Length (nt)	674
N50	983
N70	552
N90	289
GC (%)	39.39
**Transcripts annotated in databases**	
Nr	30,430
Nt	21,301
Swiss-Prot	15,876
KEGG	16,267
COG	16,700
GO	11,530
InterPro	17,980

### Sequence annotation

**Fig 2:**
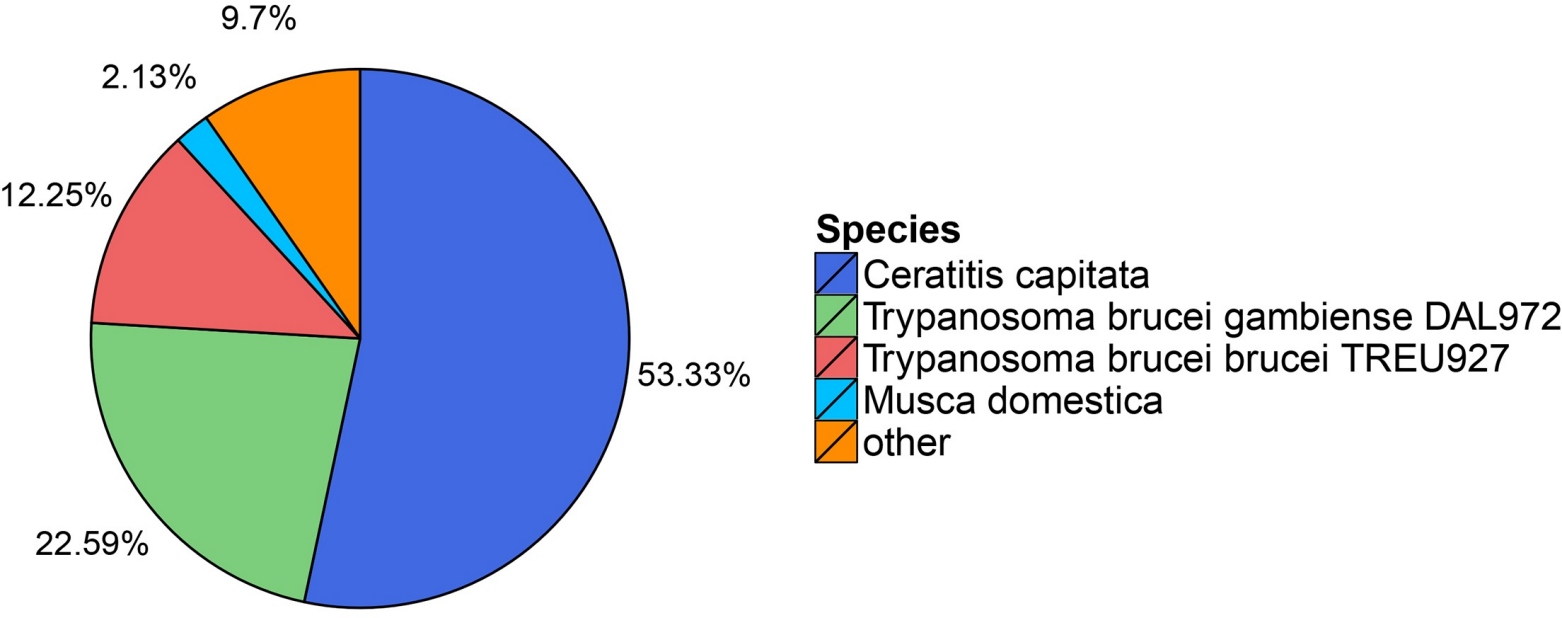
Species distribution of the top BLASTX matches to the *P*. *utilis* alimentary canal unigenes.

To functionally annotate the *P*. *utilis* midgut transcriptome, 34,564 (75.16%) unigene sequences were successfully aligned to the NCBI protein databases using BLASTX with an E-value cut-off of 1e-5. Among these unigenes, 30,430 (66.17%), 21,301 (46.32%), 15,876 (34.52%), 16,267 (35.37%), 16,700 (36.32%), 17,980 (39.10%), and 11,530 (25.07%) had highly significant matches to known proteins in the nr, nt, SwissProt, KEGG, KOG, InterPro, and GO databases, respectively (Table 1). The majority of the unigene sequences (53.33%) had best matches to sequences in the nr database from *Ceratitis capitata*, followed by *Trypanosoma brucei gambiense* DAL972 (22.59%), *Trypanosoma brucei brucei* TREU927 (12.25%), and *Musca domestica* (2.13%) (Fig 2). These results suggest that the genetic relationship between *P*. *utilis* and *C*. *capitata* is relatively close compared to other species.

**Fig 3:**
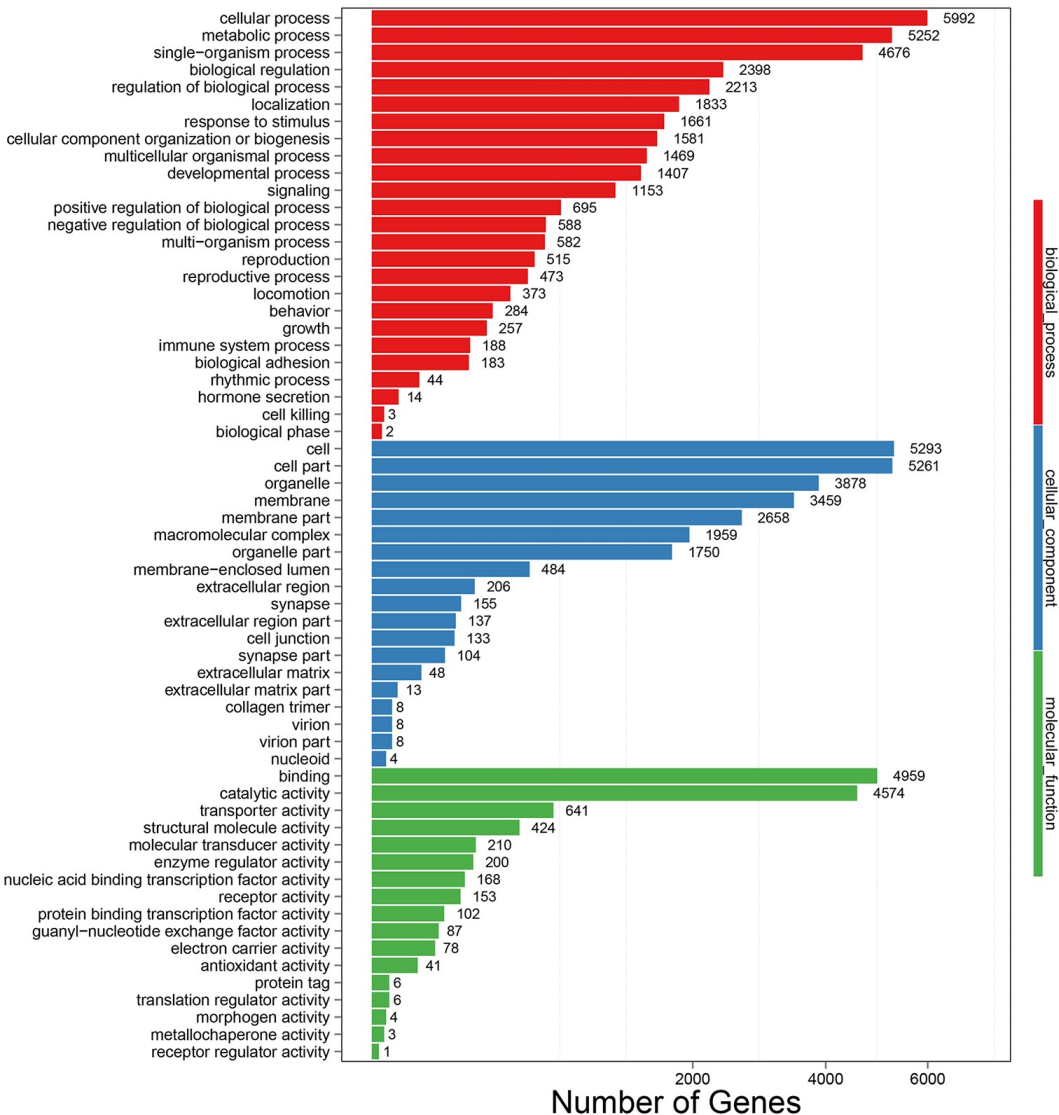
Gene Ontology (GO) classification of *P*. *utilis* alimentary canal transcriptome unigenes.

Gene Ontology (GO) assignments were further used to predict and identify the functions of the alimentary canal unigenes of *P*. *utilis*. A total of 11,530 (25.07%) unigenes were annotated and categorized into 61 functional groups (Table 1 and Fig 3). The unigenes were assigned 68,401 total GO terms, and the GO terms were divided into three ontologies: biological process, cellular component, and molecular function, which included 33,836, 25,566, and 11,657 unigenes, respectively. In the biological process category, the three most abundant subcategories were ‘cellular process’ (5,992 unigenes), ‘metabolic process’ (5,252 unigenes), and ‘single-organism process’ (4,676 unigenes). In the cellular component category, the ‘cell’ (5,293 unigenes), ‘cell part’ (5,261 unigenes), and ‘organelle’ (3,878 unigenes) functional groups were highly represented. In the molecular function category, the ‘binding’ (4,959 unigenes) and ‘catalytic activity’ (4,574 unigenes) functional groups were the most abundant groups, in addition to the ‘receptor regulator activity’ group, which only comprised 1 unigene (Fig 3).

**Fig 4:**
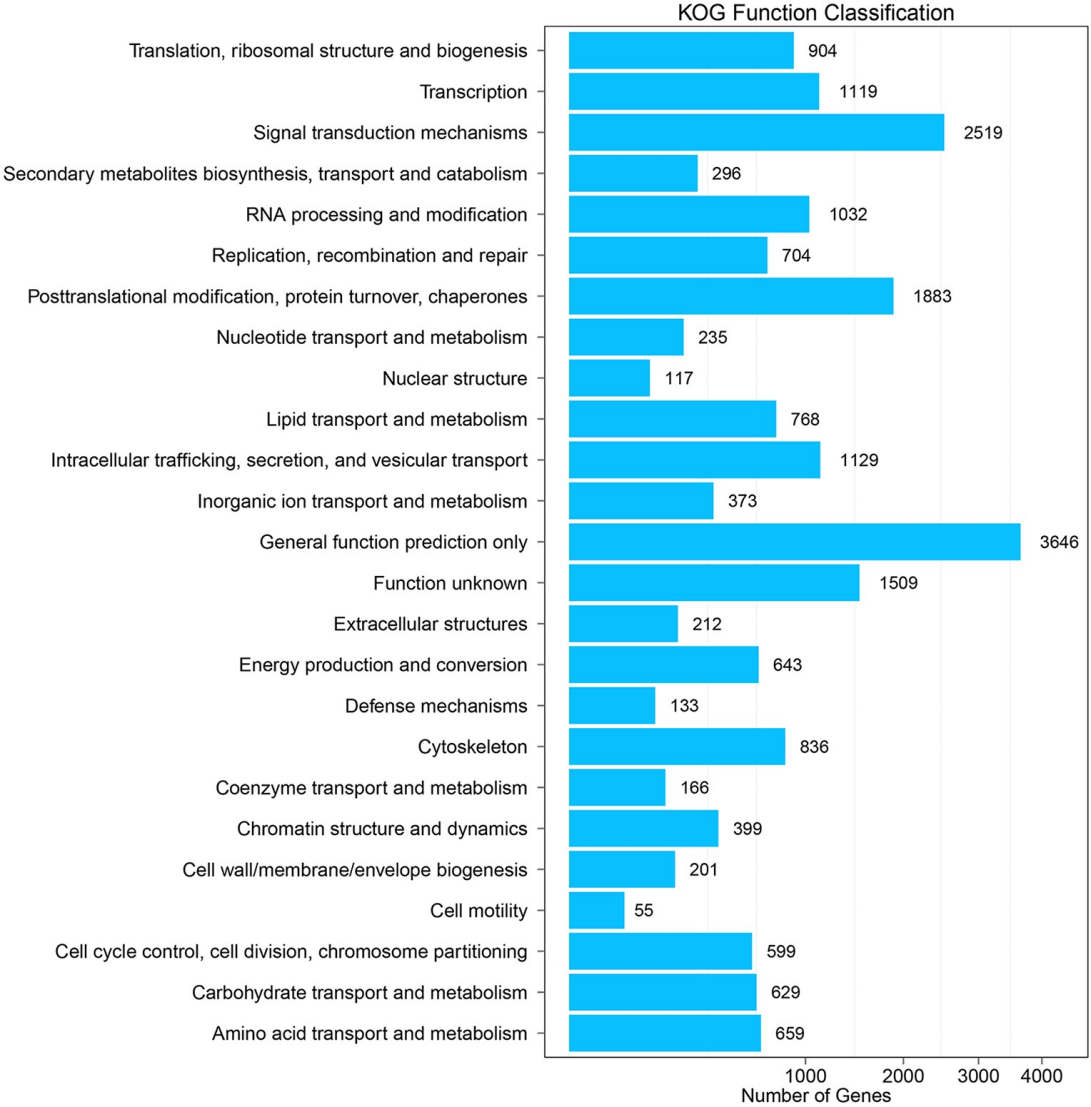
KOG categories of *P*. *utilis* alimentary canal transcriptome unigenes.

**Fig 5:**
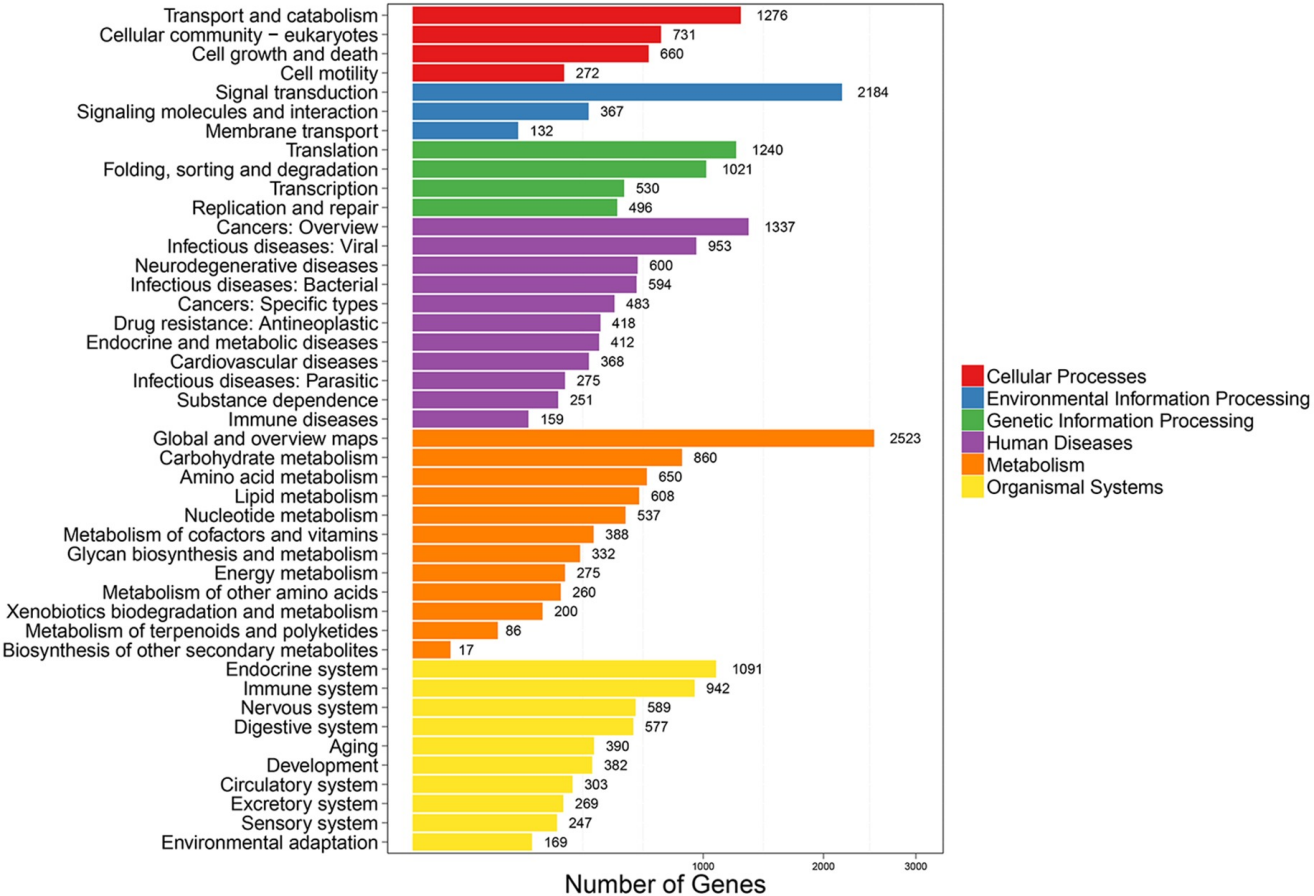
Pathway assignment of *P*. *utilis* alimentary canal unigenes based on KEGG classifications.

To further improve the annotations of the transcripts, the *P*. *utilis* alimentary canal unigenes were aligned to the KOG database to predict and classify potential functional groups. A total of 16,700 (36.32%) unigenes were annotated and classified into 25 KOG categories (Table 1 and Fig 4). ‘General function prediction only’ (3,646, 21.83%) was the largest KOG group, followed by ‘signal transduction mechanisms’ (2,519, 15.08%), ‘posttranslational modification, protein turnover, and chaperones’ (1,883, 11.28%), ‘function unknown’ (1,509, 9.048%), ‘transcription’ (1,119, 6.7%), and ‘RNA processing and modification’ (1,032, 6.17%). Other categories contained less than 1,000 unigenes, and the smallest group was ‘cell motility,’ which only comprised 55 (0.32%) unigenes. Pathway analysis was then conducted with the KEGG database to investigate the biological pathways that were associated with the unigenes. In total, 16,267 (35.37%) unigenes were mapped to KEGG Orthology (KO) terms and assigned to 323 pathways that belonged to six categories (Fig 5). ‘Global and overview maps’ (2,523, 15.5%) was the most abundant category, and ‘signal transduction’ (2,184, 13.43%) was the second largest category among the unigenes.

### Identification and expression of glutathione S-transferase genes

**Fig 6:**
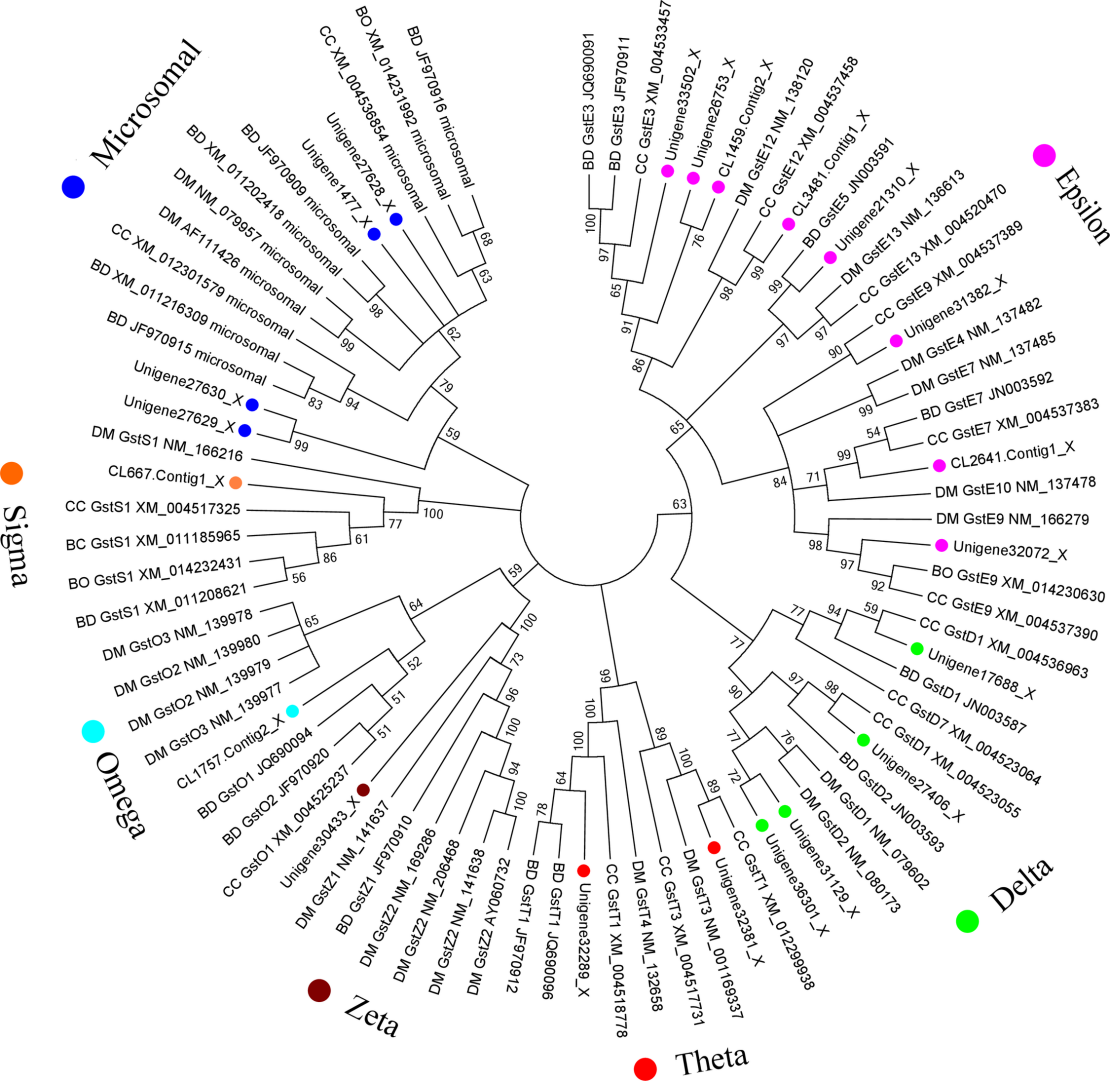
Neighbor-joining phylogenetic tree of glutathione S-transferase (GST) genes from the *P*. *utilis* alimentary canal (●) and other insects. BD, *Bactrocera dorsalis*. CC, *Ceratitis capitata*. DM, *Drosophila melanogaster*. BO, *Bactrocera oleae*. Numbers at each branch node represent bootstrap values.

A total of 22 unigenes that encoded putative GSTs were identified in the *P*. *utilis* alimentary canal transcriptome dataset using Geneious and NCBI BLASTX. Of these, 21 unigenes were chosen for further analysis after removing overly short sequences (S1 Table). Phylogenetic analysis of these GST unigenes indicated that they belonged to seven families, including the epsilon (8), delta (4), theta (2), zeta (1), omega (1), sigma (1), and microsomal (4) families, where the epsilon family was the largest and comprised 38.09% of all the GSTs (Fig 6).

**Fig 7:**
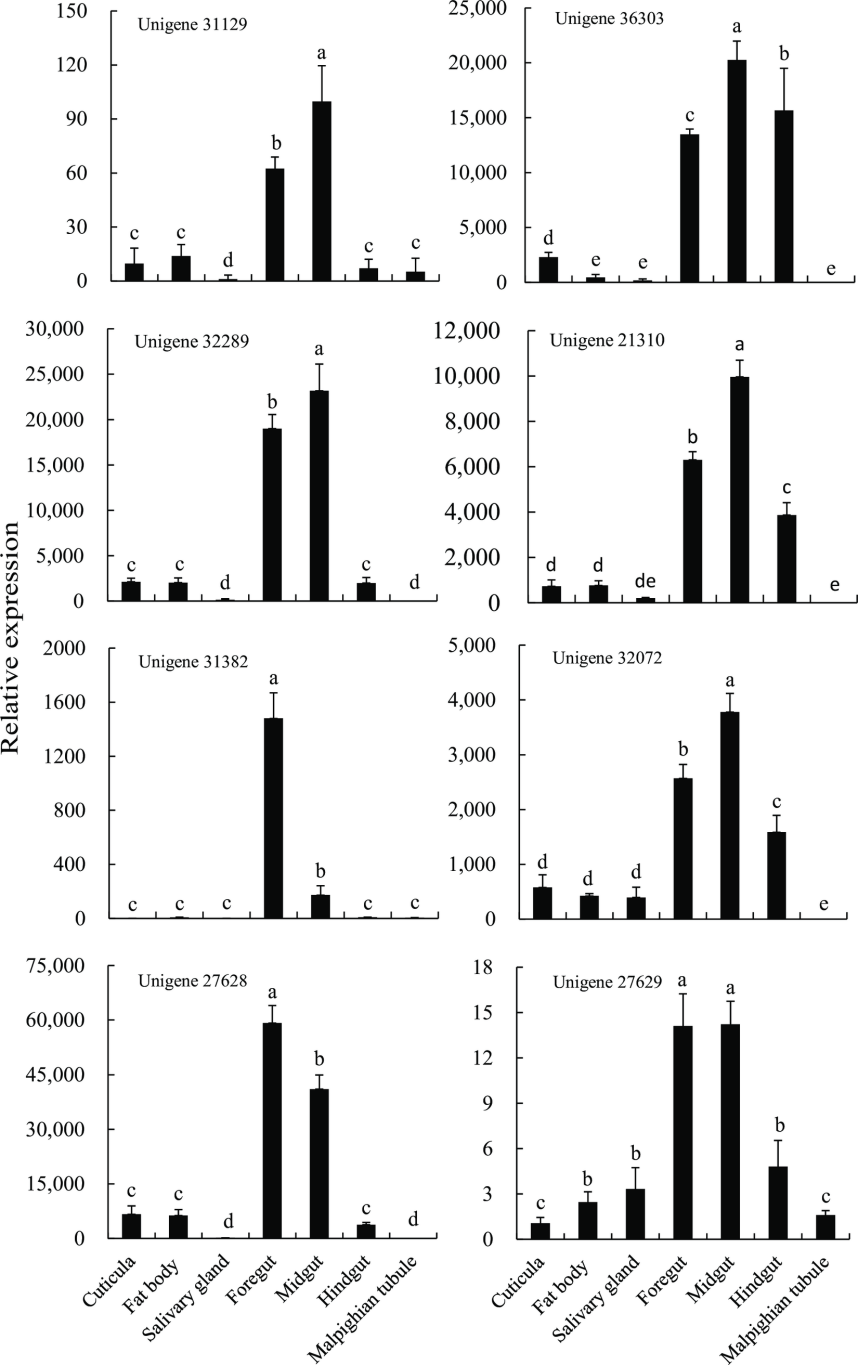
Expression of *GST* genes of *P*. *utilis* in different tissues.

Based on the FPKM value and the open reading frame length, two delta class GSTs (unigene 31129 and unigene 36303), one theta class GST (unigene 32289), three epsilon class GSTs (unigene 21310, unigene 31382, and unigene 32072), and two microsomal class GSTs (unigene 27628 and unigene 27629) were selected for qPCR expression analysis. Expression was assessed in different tissues and at different developmental stages. Four of the unigenes from the insect-specific family (unigene 31129, unigene 36303, unigene 21310, and unigene 32072), in addition to unigene 32289, were most highly expressed in midgut tissues, indicating that these genes may play an important role in the detoxification of exogenous substances. Two GSTs (unigene 31382 and unigene 27628) were most highly expressed in the foregut, suggesting that these genes may be involved in the detoxification of xenobiotics, and that the foregut tissue is an important site for detoxification. In addition, unigene 27629 was abundantly expressed in both the foregut and midgut (Fig 7). Overall, these results demonstrate that the foregut and midgut are the primary sites for detoxification within *P*. *utilis* larvae.

**Fig 8:**
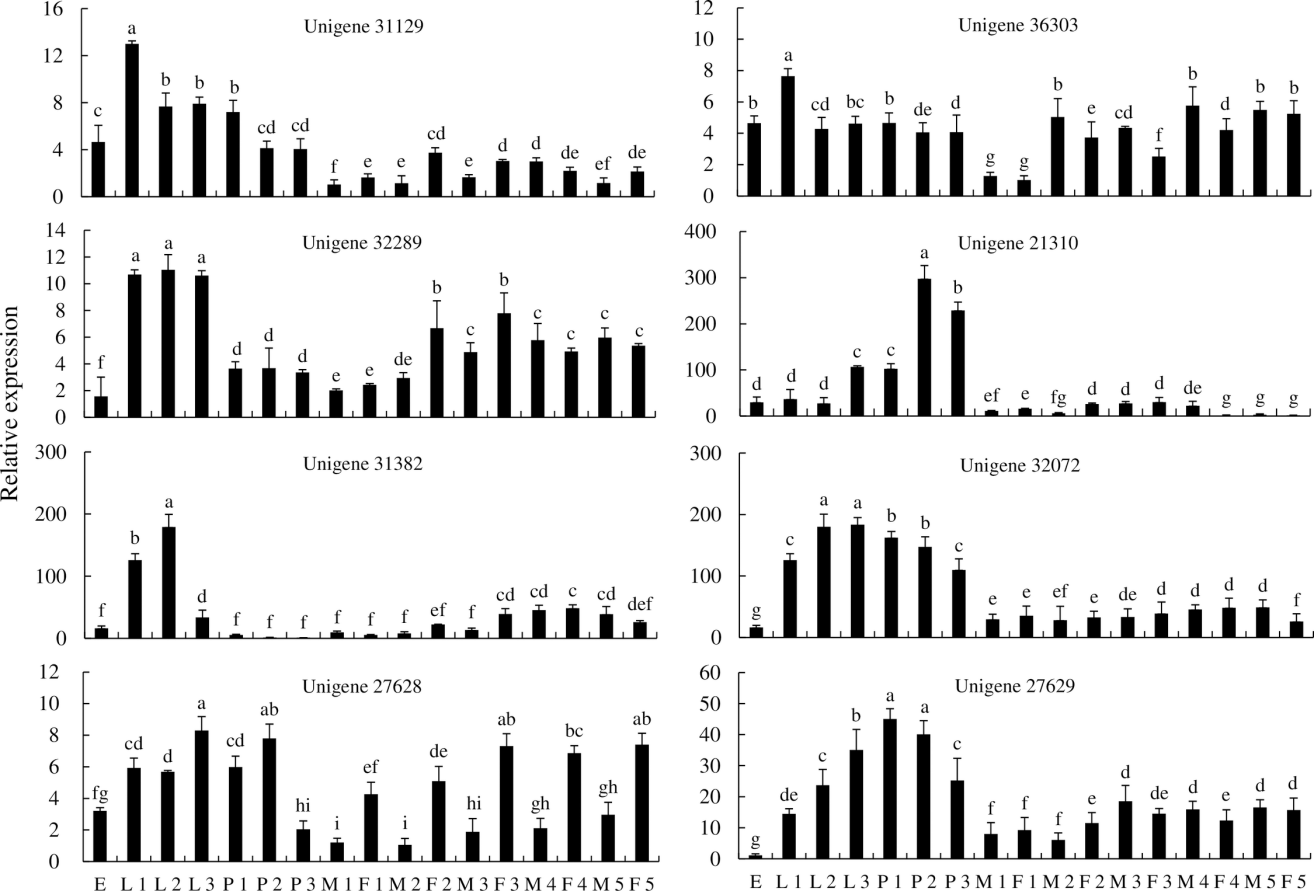
Expression of *GST* genes of *P*. *utilis* at different development stages. E, egg; L 1, first instar larvae; L 2, second instar larvae; L 3, third instar larvae; P 1, one-day pupae; P 2, two-day pupae; P 3, three-day pupae; M 1, one-day male adult; F 1, one-day female adult; M 2, two-day male adult; F 2, two-day female adult; M 3, three-day male adult; F 3, three-day female adult; M 4, four-day male adult; F 4, four-day female adult; M 5, five-day male adult; and F 5, five-day female adult.

The expression levels of unigene 31129 and unigene 36303 in first instar larvae were higher than in other developmental stages (Fig 8). The expression levels of unigene 32289 were significantly higher in the first to third larval stages compared to the other developmental stages, and the expression at the pupa and adult stages was significantly higher than in the eggs. Furthermore, the relative expression of unigene 32289 in female adults one and three days after eclosion was higher than in male adults, while the expression levels showed the opposite trend for adults four and five days after eclosion. The relative expression levels of unigene 21310 in one and two day pupae were significantly higher than in other developmental stages. Unigene 31382 was expressed at the highest levels in third instar larvae. The levels of unigene 32072 expression in second instar larvae and third instar larvae were significantly higher than in eggs, first instar larvae, pupae, and adults. The expression levels of the two microsomal GSTs (unigene 27628 and unigene 27629) varied in different development stages. For example, the relative expression of unigene 27628 in the third instar larval stage, white pupae, and grey-black pupae was significantly higher than that in eggs. However, after emergence, the relative expression gradually increased with the duration of adult age. The relative expression in male adults were significantly lower than those in female adults. Likewise, the relative expression levels of unigene 27629 in larvae, pupae, and adults were significantly higher than in eggs. Moreover, expression was highest in the white pupae and grey-black pupae, while the relative expression in male and female adults did not exhibit any obvious trends (Fig 8). Overall, the results indicate that the GSTs were mainly expressed in larvae and pupae.

### Identification and expression of cytochrome P450 genes

**Fig 9:**
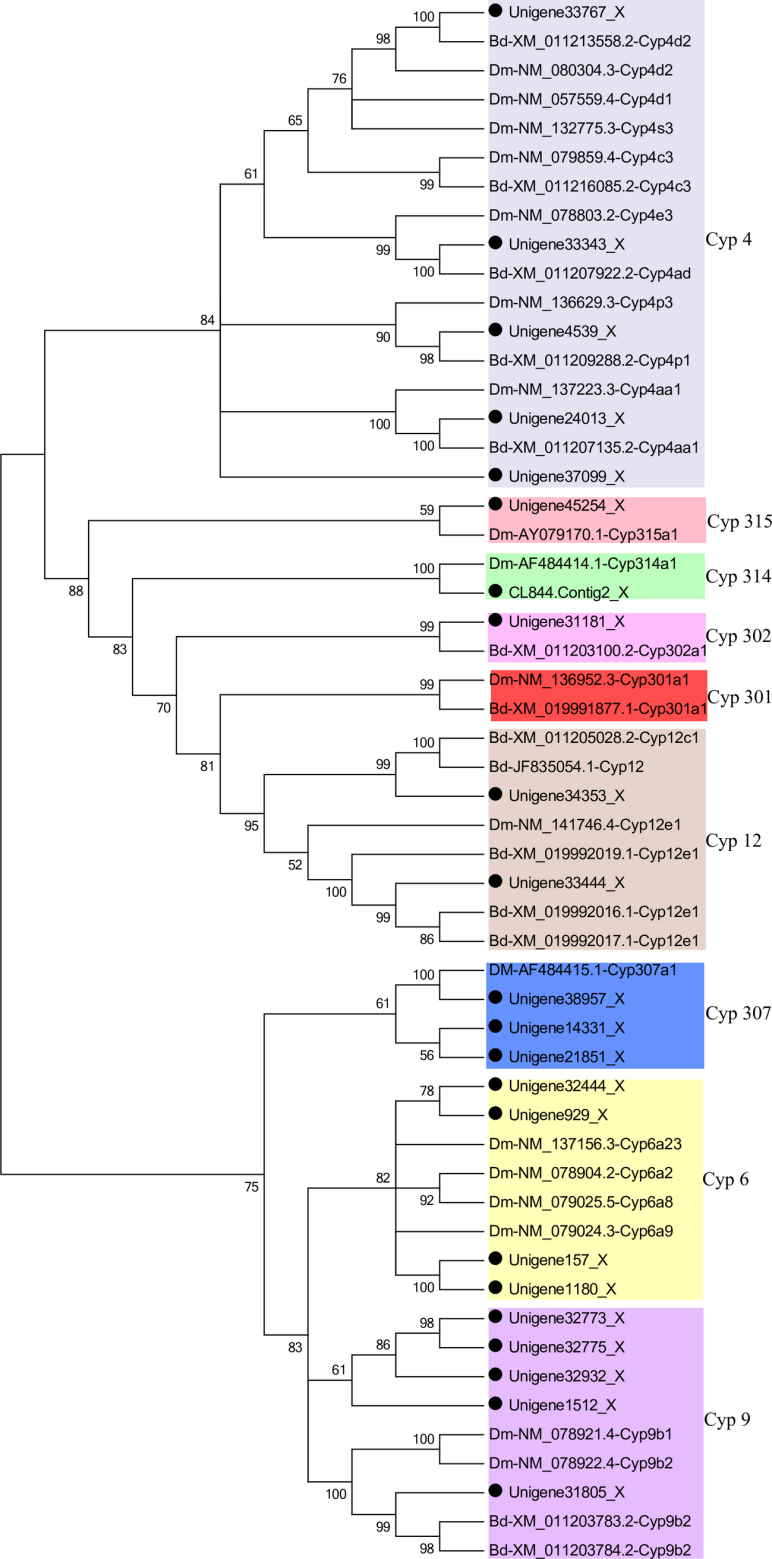
Neighbor-joining phylogenetic tree of *P450* genes from the *P*. *utilis* alimentary canal (●) and other insects. Bd, *Bactrocera dorsalis*. Dm, *Drosophila melanogaster*. Numbers at each branch node represent bootstrap values.

Analysis of the *P*. *utilis* alimentary canal transcriptome data set with Geneious and NCBI BLASTX identified 36 putative *P450* genes. After removing short sequences, 22 unigenes that encode P450s were identified (S2 Table). Based on the phylogenetic analysis, the *P450* unigenes comprised eight families, with five belonging to the Cyp 4 family, five to the Cyp 9 family, and four to the Cyp 6 family, while three and two genes belonged to the Cyp 307 and Cyp 12 families, respectively. Lastly, a single gene was identified in each of the Cyp 315, Cyp 314, and Cyp 302 families (Fig 9).

**Fig 10:**
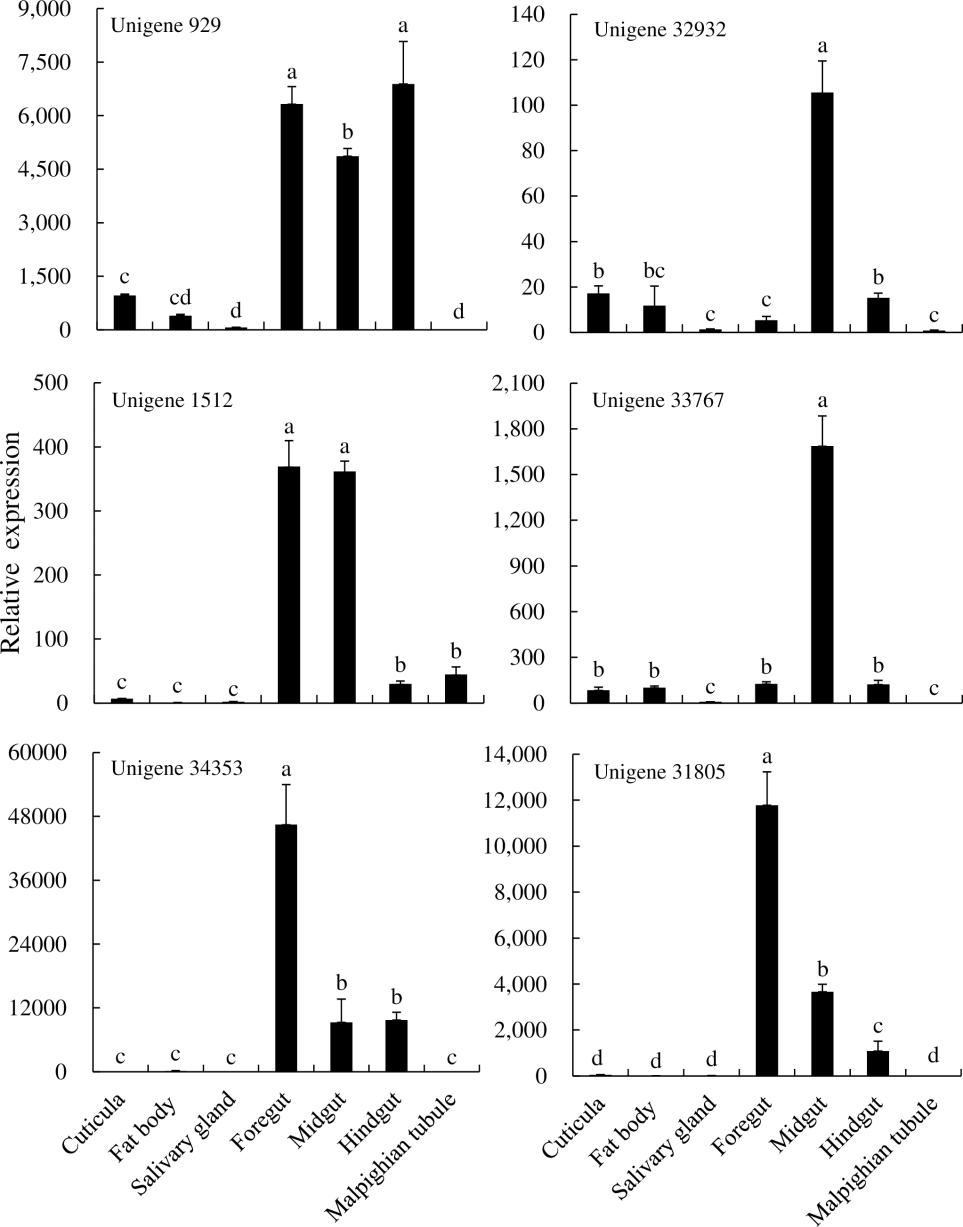
Expression of *P450* genes of *P*. *utilis* in different tissues.

Six unigenes were selected based on their FPKM values and the open reading frame length for expression analysis by qPCR in different tissues and at different developmental stages. Unigene 929 was expressed at significantly higher levels in the foregut, midgut, and hindgut compared to the other four tissues. Unigenes 32932 and 33767 were expressed at their highest levels in the midgut. In addition, the expression levels of unigene 1512 were significantly higher in the foregut and midgut compared to the other tissues. Unigenes 34353 and 31805 expression levels were the highest in the foregut than in other tissues. Taken together, these results demonstrated that the foregut and midgut of *P*. *utilis* larvae are the primary sites for P450-mediated detoxification (Fig 10).

**Fig 11:**
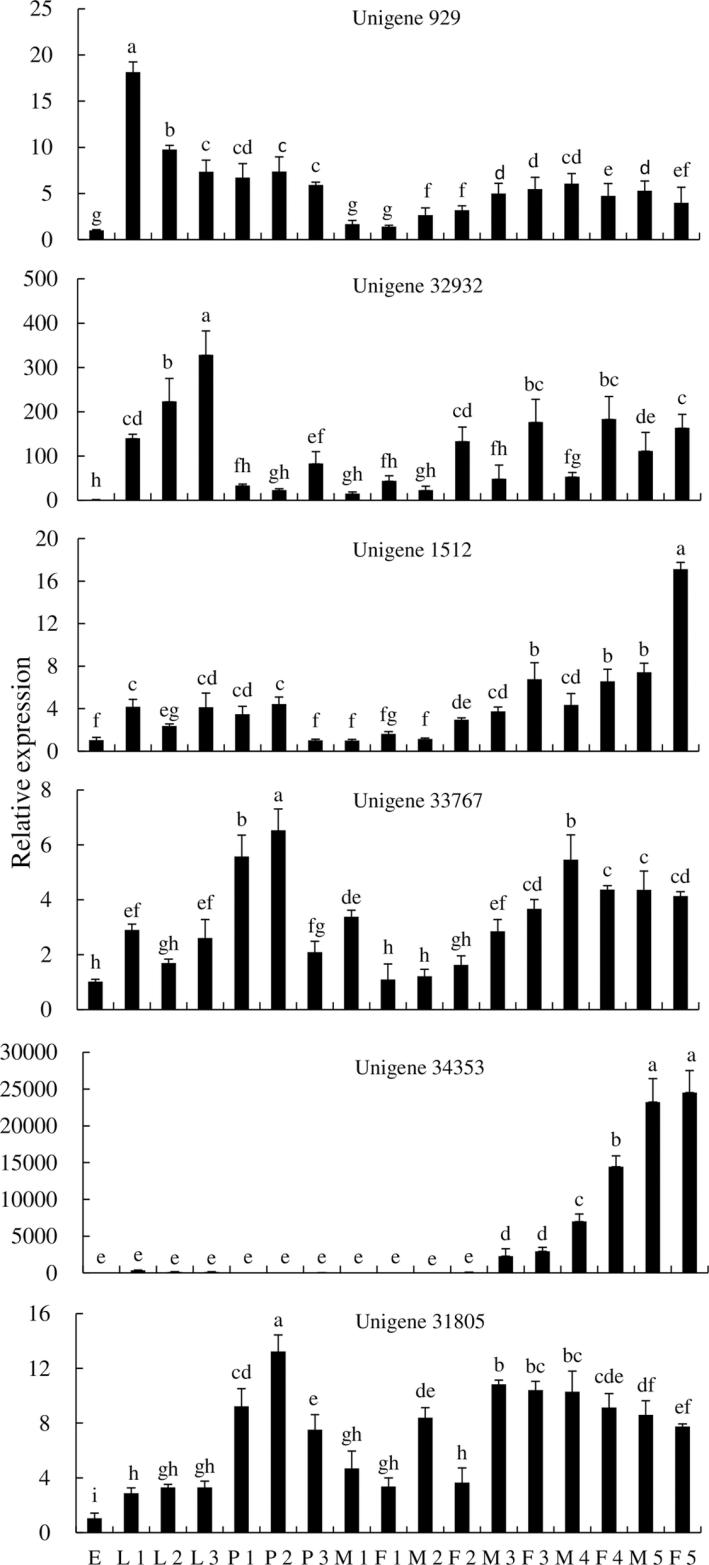
Expression of *P450* genes of *P*. *utilis* at different developmental stages. E, egg; L 1, first instar larvae; L 2, second instar larvae; L 3, third instar larvae; P 1, one day pupae; P 2, two day pupae; P 3, three day pupae; M 1, one day male adult; F 1, one day female adult; M 2, two day male adult; F 2, two day female adult; M 3, three day male adult; F 3, three day female adult; M 4, four day male adult; F 4, four day female adult; M 5, five day male adult; F 5, five days female adult.

The expression patterns of P450s in different developmental stages of *P*. *utilis* indicated that unigene 929 was expressed at all life stages (eggs, 1–3 instar larvae, pupae, male and female adults), while the relative expression level was highest in the first instar larvae. Unigene 32932 was expressed at very low levels in eggs and then increased gradually from the first instar larvae to the highest third instar larvae. Unigene 32932 expression was lower in male adults than in female adults over the same developmental period. Unigene 1512 was expressed in every developmental stage of *P*. *utilis*, with maximum expression in five-day male adults. The expression of unigene 33767 in three-day pupae was significantly higher than in other developmental stages, and the expression levels in larvae were also higher than in eggs. Unigene 34353 was highly expressed in two- to five-day adults, while it was expressed at very low levels in the eggs, larvae, pupae, and one-day adults. Lastly, the expression level of unigene 31805 in two-day pupae was significantly higher than in other developmental stages (Fig 11).

### Identification and expression analysis of carboxylesterase genes

**Fig 12:**
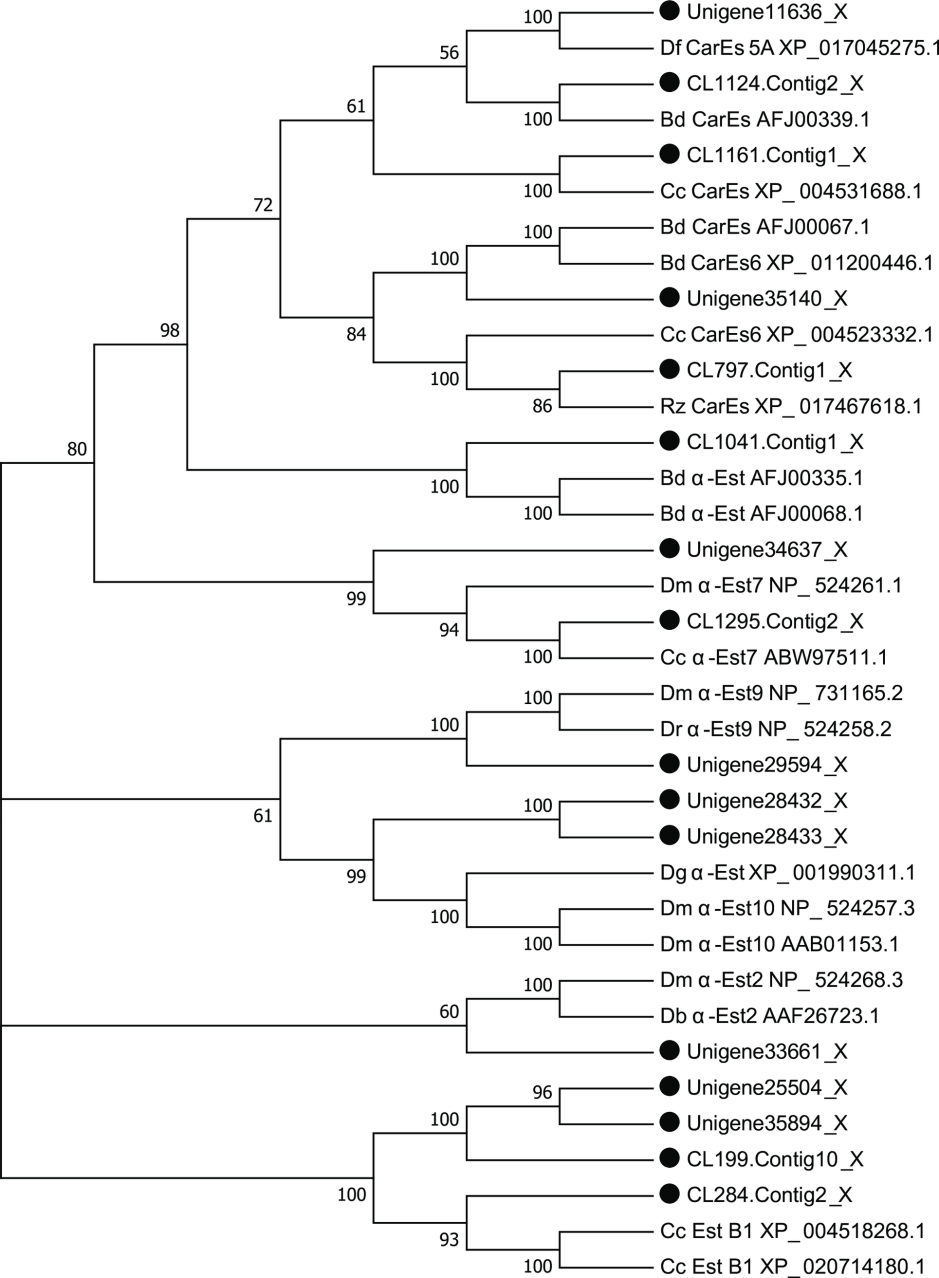
Neighbor-joining phylogenetic analysis of *CarE* genes from the *P*. *utilis* alimentary canal (●) and other insects. BD, *Bactrocera dorsalis*. CC, *Ceratitis capitata*. DM, *Drosophila melanogaster*. Rz, *Rhagoletis zephyria*. Df, *Drosophila ficusphila*. Dg, *Drosophila grimshawi*. Numbers at each branch node represent bootstrap values.

A total of 17 unigenes encoding carboxylesterases (CarEs) were identified in the *P*. *utilis* larval alimentary tract transcriptome using Geneious and NCBI-BLASTX. After removing overly short sequences, 16 unigenes were chosen for phylogenetic analysis, along with genes from other insects that were available in public databases (S3 Table). The phylogenetic analysis indicated that seven of the sequences identified here had high homology with a-esterase, which is an important component of CarEs (Fig 12).

**Fig 13:**
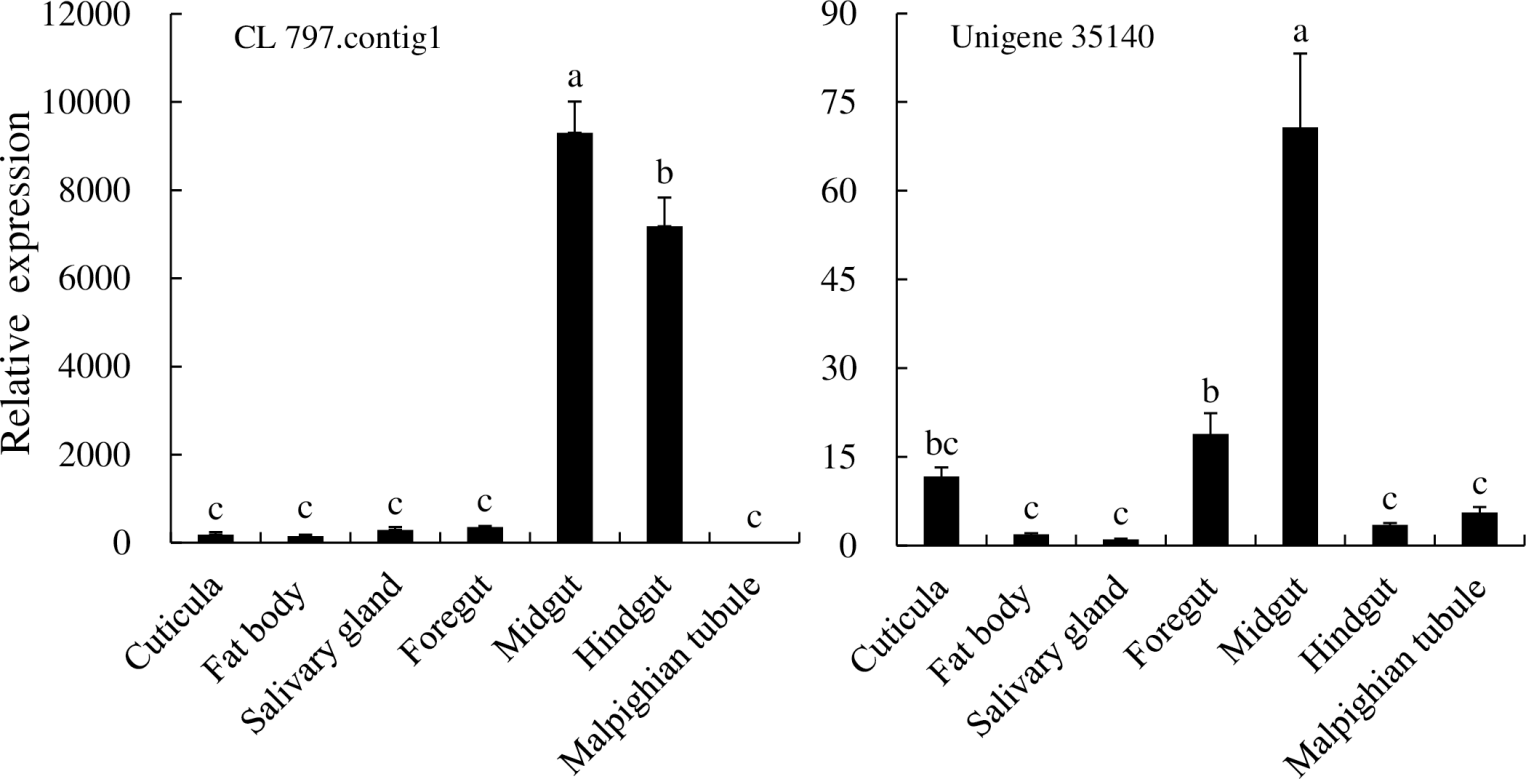
Expression of *CarE* genes of *P*. *utilis* in different tissues.

**Fig 14:**
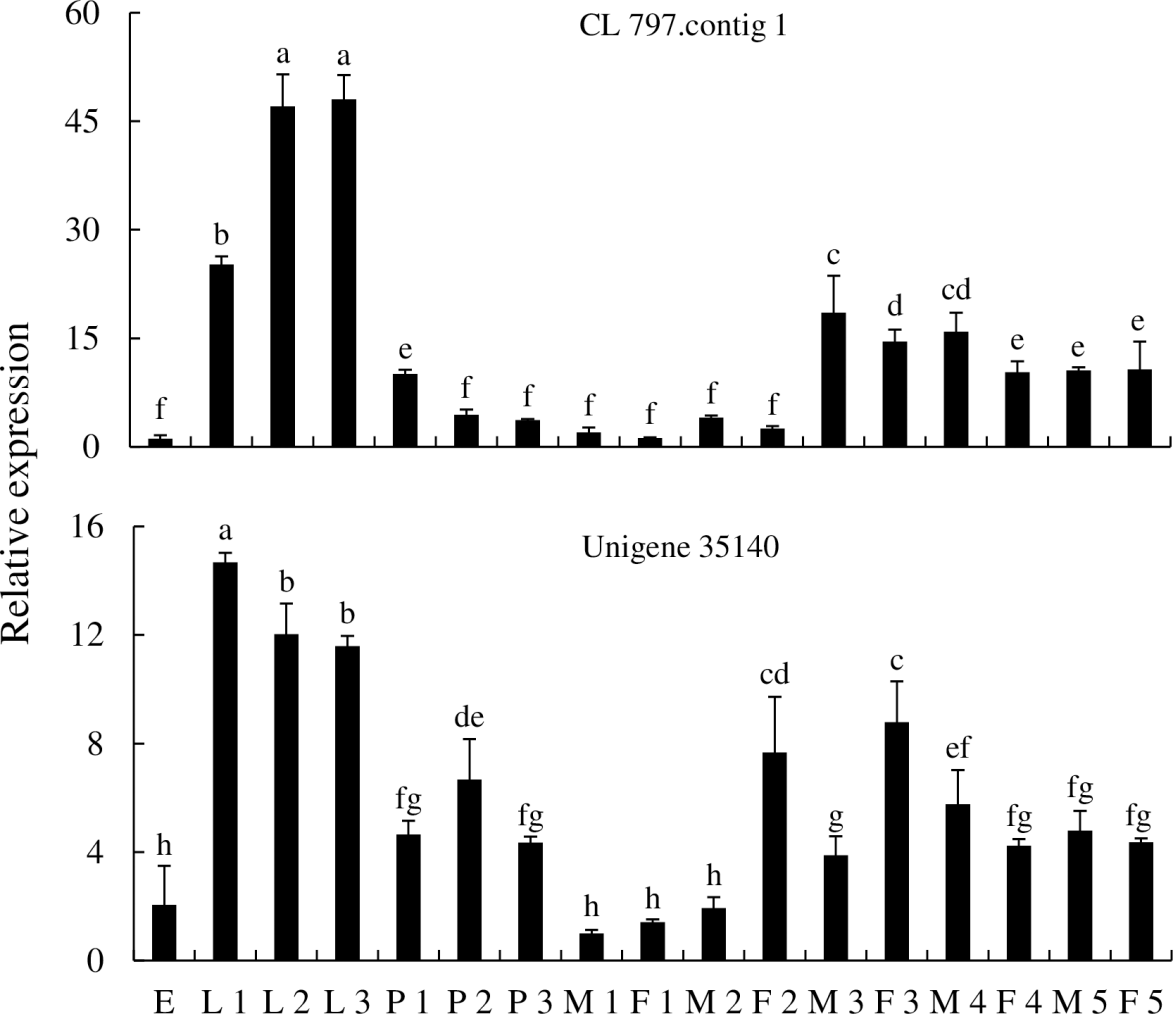
Expression of *CarE* genes of *P*. *utilis* at different developmental stages. E, egg; L 1, first instar larvae; L 2, second instar larvae; L 3, third instar larvae; P 1, one day pupae; P 2, two day pupae; P 3, three day pupae; M 1, one day male adult; F 1, one day female adult; M 2, two day male adult; F 2, two day female adult; M 3, three day male adult; F 3, three day female adult; M 4, four day male adult; F 4, four day female adult; M 5, five day male adult; F 5, five days female adult.

Two *CarE* genes (CL 797.contig and unigene 35140) were selected for expression analysis in different tissues and at different developmental stages. CL 797.contig was predominantly expressed in the larval midgut and hindgut, while it was expressed at lower levels in the Malpighian tubules. Unigene 35140 was expressed in the cuticula, fat body, salivary gland, hindgut, and Malpighian tubules, but the highest expression levels were in the midgut (Fig 13). In different developmental stages, the CL 797.contig was mainly expressed in larvae and three-day adults, with the highest expression in the second and third instar larvae. Unigene 35140 was mainly expressed in the larvae, pupae, two day female adults, and the three day adults (both male and female) (Fig 14).

## Discussion

*P*. *utilis* is a phytophagous insect that feeds specifically on the poisonous plant *E*. *adenophorum* and consequently plays an important role in the control of this weed. Many studies have addressed the ecology and biology of *P*. *utilis*, but lack of genetic data is a significant obstacle to better understanding the mechanisms of *P*. *utilis* adapting to its poisonous host plant [17]. Here, we generated a *P*. *utilis* larvae alimentary tract transcriptome via Illumina sequencing that comprised 37,726,584 raw reads and 45,985 unigenes. A previous analysis of whole *P*. *utilis* larvae generated fewer reads (13,333,334) but comprised a greater number (58,562) of unigenes [48]. The unigene data described here, and their functional annotations, provide valuable resources for further molecular studies of *P*. *utilis*.

GSTs comprise a superfamily involving with the metabolism of many endogenous and exogenous toxic compounds, including insecticides, fungicides, herbicides, and deleterious plant secondary substances [33, 54, 55]. It participates in relieving oxidative stress and regulate the biosynthesis of hormones and intracellular transport [56, 57]. The GSTs of *Hyphantria cunea* moths play a key role in degrading *Ginkgo biloba* plant secondary metabolites, such as ginkgo flavonoids and ginkgolides [27]. In *Oedaleus asiaticus* grasshoppers, the activities of GSTs are positively related to levels of secondary plant substances [58]. Furthermore, several insects, including *Acyrthosiphon pisum*, *Myzus persicae*, and *Sitobion avenae*, use GSTs to metabolize deleterious plant secondary metabolites [59, 60]. The GST superfamily is divided into at least seven major subclasses in insects: the delta, epsilon, omega, sigma, theta, zeta, and microsomal classes. The delta and epsilon classes are specific to insects [57, 61], while the others are distributed more broadly. The delta, epsilon, omega, and zeta class GSTs play dominant roles in the metabolism of endogenous and exogenous compounds within insects [62]. For example, the delta and epsilon GSTs are directly involved in pesticide resistance [36, 63]. Some studies have suggested that the omega class GSTs are related to the elimination of S-thiol adducts from proteins [62], and may also be associated with oxidative stress responses [62, 64]. Further, the sigma- and zeta-class GSTs may be involved in protection against oxidative stress, and the latter may also be involved in pesticide resistance [62, 65]. Likewise, the microsomal GSTs may be involved in protection against oxidative stress and the removal of toxic xenobiotics [62].

The GST superfamily has been identified in many insect species. For instance, 41, 27, 18, 28, and 36 GSTs have been identified in *Drosophila melanogaster*, *Bactrocera dorsalis*, *Bactrocera minax*, *Grapholita molesta*, and *Shirakiacris shirakii*, respectively [38, 61, 35, 66]. We identified 21 GST genes in *P*. *utilis* that belong to seven of the GST classes: epsilon (8), delta (4), theta (2), zeta (1), omega (1), sigma (1), and microsomal (4). Epsilon is the most represented GST, comprising 38.09% of the total that is consistent with other dipteran insects [65, 66]. Several studies have shown that delta- and epsilon-class GSTs are associated with metabolic detoxification and adaptations to selective environmental pressures [36]. The presence of these gene families in *P*. *utilis* may be beneficial to its survival in the poisonous and hostile environment of its host plant. Eight GST genes of *P*. *utilis* were used to identify expression patterns throughout various tissues and at different developmental stages. GST gene expression was the highest in the midgut for six of the genes (four were insect-specific), while two of the GSTs were expressed at the highest levels in the foregut. These results suggest that the midgut and foregut are vital for *P*. *utilis* detoxification. The eight genes were expressed in all of the developmental stages of *P*. *utilis*, but expression was highest in larvae and pupae, and lowest in eggs. These results are consistent with those observed for *B*. *dorsalis*, *Anopheles gambiae*, *Locusta migratoria manilensis*, and *Liposcelis entomophila* (Enderlein), wherein genes exhibited developmental stage-specific expression [67–69]. The high expression of GSTs in the larval stage of *P*. *utilis* may be related to the detoxification of secondary metabolites associated with the host, *E*. *adenophorum*.

P450s comprise a large and complex superfamily of enzymes that are involved in the synthesis of hormones and the metabolism of endogenous and xenobiotic compounds. The functional diversity of P450s has contributed to the successful adaptation of insects to a variety of ecological environments [70, 71]. Multiple *P450* family genes have been identified in herbivorous insects, which are adaptations in insect–plant antagonistic evolution [72]. Previous studies have shown that the ingestion of plant material frequently induces insect P450 genes that are involved in the detoxification of plant toxins. For example, in the black swallowtail butterfly, *Papilio polyxenes*, the P450s *CYP6B1v1* and *CYP6B3v1* metabolize the furanocoumarins present in their diet [73, 74]. Further, *CYP321A1* and *CYP6B8* from the corn earworm (*Helicoverpa zea*) can metabolize xanthotoxin and angelicin [75, 76]. In *Apis mellifera*, *CYP6AS3* is involved in the detoxification of quercetin, which is a flavonol found in plant nectar [77]. Furthermore, the expression of *CYP6A8*, *CYP6D5*, *CYP6W1*, *CYP9B2*, and *CYP12D1* genes in *D*. *melanogaster* were induced by *Piper nigrum* extracts, and *CYP6A2* and *CYP6A8* expression was induced by caffeine [78, 79]. Likewise, the expression of *CYP6AB14*, *CYP321A7*, and *CYP321A9* in *Spodoptera litura* was induced by the toxic allochemicals xanthotoxin, coumarin, and flavones [80, 81]. We identified 36 *P450* genes that were expressed in the *P*. *utilis* larval alimentary tract. Most of the P450s in *P*. *utilis* were members of the Cyp4 and Cyp6 families, which is similar to other insects. In addition, *P*. *utilis* exhibited greater representation of two (CYP3 and CYP4) other families. [71, 82]. The CYP3 group is a large family of insect-specific CYP P450s, including the CYP6, CYP9, CYP28, and CYP308-310 families [82].

To explore the function of *P450* genes in the *P*. *utilis* larval alimentary tract, the expression patterns of six unigenes (unigene 929, unigene 32932, unigene 1512, unigene 33767, unigene 34353, and unigene 31805) were determined in different tissues of third instar larvae and at different developmental stages using qPCR. Unigene 929 was primarily expressed in the larval foregut, midgut and hindgut, while two P450s (unigene 32932 and unigene 33767) were mainly expressed in the larval midgut. Unigene 1512 was mainly expressed in the larval foregut and midgut, whereas unigenes 34353 and 31805 were mainly expressed in the larval foregut. Previous studies have shown that the midgut and fat body of insect larvae are the primary regions for detoxification, where ingested plant allelochemicals can be efficiently detoxified before food absorption [81, 82]. The expression levels of *CYP6B48*, *CYP6B58*, *CYP6AB14*, *CYP9A40*, and *CYP321B1* were highest in the midgut and fat body of *S*. *litura* [80–84]. Likewise, *CYP321A7*, *CYP321A8*, *CYP321A9*, and *CYP321A10* in *S*. *frugiperda*, and *CYP6CV1* in *Cnaphalocrocis medinalis* were also highest in the midgut and fat bodies [21, 85]. In contrast, *CYP321A1* in *H*. *zea* [86] and *CYP9A38* in *C*. *medinalis* were mainly expressed in the midgut [21]. In addition, most P450s of *D*. *melanogaster* are expressed in the larval midgut, Malpighian tubules, and the fat body, and these tissues are critical for the metabolism and detoxification of xenobiotics [31]. *CYP367s* in *P*. *xylostella* and *CYP6BQ9* in *Tribolium castaneum* were primarily expressed in the head, suggesting a potential role in either olfaction or detoxification in this area [87, 88]. The P450s of *P*. *utilis* were mainly expressed in the foregut and midgut, indicating that these areas are the main tissues responsible for the metabolism and detoxification of xenobiotics for *P*. *utilis* larvae. The highest expression levels of unigene 929, unigene 32932, unigene 1512, unigene 33767, unigene 34353, and unigene 31805 were detected in the first instar larvae, third instar larvae, three day pupae, and adults (male and female) after eclosion for five days. However, the expression levels of these genes varied between the life stages of *P*. *utilis*. The expression of unigene 929 in *P*. *utilis* larvae was significantly higher in the first instar larvae than in the other developmental stages. Likewise, unigene 32932 expression was significantly higher in third instar larvae than in other developmental stages. The expression level of unigene 1512 was significantly higher in female adults five days after eclosion compared to the other developmental stages. The expression levels of unigenes 33767 and 31805 were significantly higher in the two-day old pupae. Unigene 34353 expression was significantly higher in both male and female adults five days after eclosion. Likewise, *CYP6CV1* was most highly expressed in the fourth and fifth instar larvae, as well as in adults of *C*. *medinalis*, although *CYP9A38* was expressed at the highest level in the third and fourth instar larvae [72]. In *P*. *xylostella*, the expression levels of *CYP4G78*, *CYP301B1*, and *CYP315A1* were highest in the pupae, while *CYP6BF1*, *CYP6CV2*, and *CYP6BG3* were highest in the larvae, and *CYP6BD11*, *CYP6CN1*, *CYP314A1*, and *CYP4G77* were highest in adults, particularly in males [87]. Further, the highest expression levels of *CYP321A7* and *CYP321A9* were observed in the fifth and sixth instar larvae of *S*. *ilitura* [81]. These studies suggest that the levels of individual *P450* expression can vary among life stages [72].

CarE is a multifunctional superfamily enzyme that plays important roles in the hydrolysis of neurotransmitters, detoxification, pheromone degradation, and regulation of development [89, 90]. It is also one of the most important detoxification enzymes in insects and is not only related to insect toxin resistance (i.e., to organophosphorus, carbamate, and pyrethroid) but also to the detoxification of plant secondary substances, wherein it can be induced by plant secondary metabolites [21, 91]. For instance, the activity of CarE in *Helicoverpa armigera* larvae was significantly induced by quercetin [91]. Likewise, CarE activity was significantly higher in *Lymantria dispar* after being fed phenolic glycoside [91, 92]. The detoxification activities of CarEs were highest in *Oedaleus asiaticus* when feeding *Artemisia frigida* (which have high levels of secondary compounds) compared to *O*. *asiaticus* feeding grass with low levels of secondary compounds [58].

Numerous CarEs have been identified from different insect species. For example, 36, 50, 46, 12, and 76 CarEs have been identified in *S*. *shirakii*, *Anopheles sinensis*, *Grapholita molesta*, *B*. *dorsalis*, and *Bombyx mori*, respectively [66, 71, 35, 38, 93]. In this study, 17 CarEs were identified in the transcriptome of the *P*. *utilis* larvae alimentary tract, and two (CL 797.contig and unigene 35140) *CarE* genes were used to explore expression patterns in different tissues and at different development stages. The CL 797.contig gene was predominantly expressed in the larval midgut and hindgut, whereas unigene 35140 was mainly expressed in the larval midgut. These data may further suggest that the larval midgut and hindgut may play important roles in the detoxification of secondary metabolites. CarEs were highly expressed not only in the midgut of *B*. *mori* but also in the Malpighian tubules, integument, head, fat body, and testis, indicating that they may protect *B*. *mori* from xenobiotic damage and participate in the metabolism of plant secondary compounds and other substances [93]. CarEs were expressed differentially in different tissues of *Reticulitermes flavipes*, although expression was concentrated in the midgut, Malpighian tubes, and fat bodies [94, 95]. Furthermore, *BdCAREB1* of *B*. *dorsalis* was significantly expressed in the fat body [96]. In addition to spatial differentiation, the expression of *CarE* genes in different developmental stages of insects has been observed. The expression levels of two CarEs (*Pxae22* and *Pxae31*) were highest in the 4th instar larvae of *Plutella xylostella* [42]. In *B*. *dorsalis*, *BdCAREB1* was most highly expressed in the third instar larvae, while low expression was observed in eggs and pupae [96]. The CL 797.contig 1 gene in *P*. *utilis* was primarily expressed at stages of larvae and three-day adults, with its highest expression in the second and third instar larvae. In contrast, the unigene 35140 of *P*. *utilis* was primarily expressed in the larvae, pupae, two-day female adults, and three-day adults (male and female). Thus, the expression of CarEs in different developmental stages changes with growth and development. These results indicate that the CL 797.contig 1 and unigene 35140 genes may be involved in the detoxification of toxic compounds within *E*. *adenophorum* and may also participate in other physiological functions of adults.

## Supporting information

S1 Table Unigene sequences for the glutathione s-transferases identified in the *Procecidochares utilis* transcriptome. (XLSX)

S2 Table Unigene sequences for cytochrome oxidase P450s identified in the *Procecidochares utilis* transcriptome. (XLSX)

S3 Table Unigene sequences for carboxylesterases identified in the *Procecidochares utilis* transcriptome. (XLSX)
